# Syntheses of Enantiopure Aliphatic Secondary Alcohols and Acetates by Bioresolution with Lipase B from *Candida antarctica*

**DOI:** 10.3390/molecules17088955

**Published:** 2012-07-26

**Authors:** Hercules V. Ferreira, Lenilson C. Rocha, Richele P. Severino, André L. M. Porto

**Affiliations:** 1 Institute of Chemistry of São Carlos, University of São Paulo, Av. Trabalhador São-carlense, 400, 13560-970, São Carlos, SP, Brazil; Email: hercules_quimica@hotmail.com (H.V.F.); lenilson@iqsc.usp.br (L.C.R.); richeleps@yahoo.com.br (R.P.S.); 2 Department of Chemistry, Federal University of Goias, Campus Advanced of Catalão, Av. Dr. Lamartine Pinto de Avelar, 1120, 75704-020, Catalão, GO, Brazil

**Keywords:** kinetic resolution, lipase, *Candida antarctica*, aliphatic secondary alcohols

## Abstract

The lipase B from *Candida antarctica* (Novozym 435^®^, CALB) efficiently catalyzed the kinetic resolution of some aliphatic secondary alcohols: (±)-4-methylpentan-2-ol (**1**), (±)-5-methylhexan-2-ol (**3**), (±)-octan-2-ol (**4**), (±)-heptan-3-ol (**5**) and (±)-oct-1-en-3-ol (**6**). The lipase showed excellent enantioselectivities in the transesterifications of racemic aliphatic secondary alcohols producing the enantiopure alcohols (>99% *ee*) and acetates (>99% *ee*) with good yields. Kinetic resolution of *rac*-alcohols was successfully achieved with CALB lipase using simple conditions, vinyl acetate as acylating agent, and hexane as non-polar solvent.

## 1. Introduction

The use of enzymes for organic synthesis has become an interesting area for organic and bio-organic chemists. Since many enzymes have been demonstrated to possess activity against non-natural substrates in organic media they have become widely use to carry out synthetic transformations. Hydrolases are the most frequently used enzymes due to their broad substrate spectrum and considerable stability. Additionally, many of them are commercially available and they work under mild reaction conditions and without the necessity for cofactors [1]. Enantiomerically pure alcohols and esters are key intermediates for asymmetric synthesis of pharmaceutical and agrochemical compounds [2,3]. Among the numerous synthetic methods for asymmetric synthesis, enzyme-catalysed kinetic resolution of racemic alcohols through transesterification is an attractive route. For example, the enantiomeric resolution of atenolol by lipase B from *Candida antarctica* [4]. Transesterification reactions catalysed by hydrolytic enzymes have been extensively studied in conventional nonaqueous solvents, producing excellent results [5,6,7,8,9]. In addition, the dynamic kinetic resolution (DKR) is a useful methodology for the conversion of racemic substrates to single enantiomer [10,11].

Lipases are frequently used as an asymmetric catalyst for the preparation of enantiomerically pure organic compounds [12,13]. Lipase B from *C. antarctica* (Novozym 435^®^) displayed esterification of aliphatic secondary *rac*-alcohols producing good enantiomeric purities to acetates (88.7–97.7% *ee*) and alcohols (42.8–85.5% *ee*) [14]. The lipase from *Pseudomonas fluorescens* (Amano AK) catalysed the transesterification reactions of secondary alcohols (2-butanol, 2-pentanol, 2-hexanol, 2-heptanol and 2-octanol) using vinyl acetate as acylating agent and producing moderate selectivities for the products [15].

The resolution of a racemic substrate can be achieved with a range of hydrolases including lipases and esterases. Kinetic resolution (KR) of racemic secondary alcohols with lipases has been shown the dependence between the structure of the substrate and the enantioselectivity of enzymatic transesterification in according to the empirical Kazlauskas’ rule [16].

Recently we reported the use of enzyme-catalysed transesterifications of secondary alcohols by kinetic resolution using different compounds to produce enantiomerically pure or enriched alcohols and acetates [17,18,19,20]. In this work, we report an efficient protocol for the KR of aliphatic secondary alcohols using mild conditions to produce enantiopure compounds by lipase CALB.

## 2. Results and Discussion

**Scheme 1:**
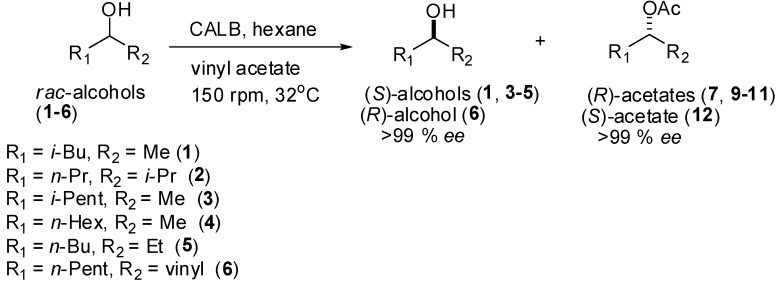
Chemoenzymatic resolution of aliphatic secondary alcohols by *C. Antarctica* lipase B.

Rocha *et al.* reported the kinetic resolution of secondary iodophenylethanols using lipase B from *Candida antarctica* (CALB) to produce alcohols and acetates with high enantiomeric excesses [17]. Efficient KR of *rac*-alcohols is successfully achieved by lipase under mild friendly conditions, vinyl acetate as acylating agent, and hexane as non-polar solvent. To extend this biocatalytic methodology, a series of aliphatic secondary alcohols **1**–**6** were used in order to obtain enantiopure products. Initially, the racemic alcohols **1**–**6** and acetates **7**–**12** were obtained with good yields by conventional methods (Scheme 1).

In general, the KR of aromatic secondary alcohols produced enantiopure compounds with CALB. However, the enzymatic kinetic resolution of aliphatic secondary alcohols did not show an effective enantioresolution, and low enantiomeric excesses are frequently obtained. In this study, our focus on the KR enabled a series of aliphatic secondary alcohols containing different groups attached to the stereogenic center. The results are summarized in Table 1. The kind of group attached to the stereogenic center is crucial for the useful kinetic resolution of secondary alcohols by CALB. The racemic substrates **1**, **3**–**4** were efficiently resolved by CALB producing the enantiopure (*S*)-alcohols (**1**, **3**–**4**) and (*R*)-acetates **7**, **9**–**10** with high enantiomeric excesses (>99% *ee*, Table 1). The studies, of the *rac*-alcohols **1**, **3**–**4** contain the methyl group attached to the stereogenic center, which were responsible for the high selectivities obtained by CALB. 

**Table 1:** Enzymatic transesterification of (±)-secondary alcohols **1**–**6** by lipase B from *Candida antarctica*^a^.

alcohols	alcohols 1–6					acetates 7–12				
	t (min)	c (%) ^b^*	*ee* (%) ^b^	ac ^b^	yield (%) ^b,d^	c (%) ^c^	*ee* (%) ^c^	ac ^c^	yield (%) ^c,^^d^	*E*
**1**	60	50	>99	*S*	40	50	>99	*R*	45	>200
**2**	60	100 ^e^	-	*-*	-	-	*-*	-	-	*-*
**3**	20	60	-	*-*	-	40	>99	-	-	-
**4**	40	50	>99 ^f^	*S*	32	50	>99	*R*	31	>200
	20	70	-	*-*	-	30	>99	-	-	-
	40	60	-	-	-	40	>99	-	-	-
**5**	60	50	>99 ^f^	*S*	40	50	>99	*R*	35	>200
	240	70	-	-	-	30	-	*-*	-	-
	420	50	>99	*S*	35	50	>99	*R*	32	>200
**6**	20	80	-	-	-	20	-	*-*	-	-
	40	55	-	-	-	45	-	*-*	-	-
	60	50	>99 ^f^	*R*	41	50	>99	*S*	50	>200

^a^ The reactions were carried out in a Erlenmeyer flask containing 10 mL of hexane (HPLC grade), 0.5 mL of vinyl acetate, 0.5 mmol of racemic alcohols **1**–**6** (0.5 mmol) and 80 mg of lipase CALB. The reaction mixture was stirred in an orbital shaker (32 °C, 150 rpm) until 50% of conversion; ^b^* concentrations of unreacted alcohols; ^c^ acetates; ^d^ isolated yield; t: time (minutes); c: conversion. ^e^ Not reacted; *ee*: enantiomeric excesses determined by chiral GC; ac: absolute configuration; *E*: enantiomeric ratio was calculated using Sih’s method [14]; ^f^ Enantiomeric excesses obtained after acetylation of alcohols.

In addition, we investigated the selectivity of lipase by increasing the steric bulk attached to the stereogenic center in other racemic alcohols. For the aliphatic alcohol **2**, with *n*-propyl and isopropyl groups linked to the stereogenic center, the enzymatic KR for the production of acetate **8** not occurred. These results confirm that the *n*-propyl and isopropyl are large groups for the KR of secondary alcohols by lipase B from *C. antarctica*. 

However, the KR for alcohols **5**–**6** were highly efficient, producing enantiopure products (>99% *ee*, Table 1). In these cases, the substituent groups attached to the stereogenic center were *n*-butyl and *n*-ethyl for **5** and *n*-pentyl and vinyl for **6**. Moreover, for the aliphatic alcohol **5** the reaction occurred slowly and the time necessary to reach 50% conversion was 7 hours (Table 1). In this investigation, the alkyl and vinyl groups were accepted by CALB, giving alcohols and acetates with excellent optical purities. In general, high selectivity for these reactions was obtained (*E* > 200). Recently, the chemoenzymatic resolution of racemic allylic alcohol **6** was described using CALB [21,22]. Lipase from *Pseudomonas cepacia* catalysed esterification of allylic alcohols with different selectivities, but in several cases not exceeding 98% *ee* to the compounds [23].

According to the empirical Kazlauskas rule, lipase stereoselectivity is mainly set by steric interactions between enzyme and substrate. The small difference in the steric bulk of ethyl and vinyl groups on aliphatic alcohols **5**–**6** compared with the methyl group on alcohols **1**, **3**–**4**, showed the occurrence of non-steric interactions, and these *rac*-alcohols reacted by lipase CALB. 

The assignment of the absolute configuration for (*S*)-alcohols **1**, **4**–**5**, (*R*)-alcohol **6**, (*R*)-acetate **7** and (*S*)-acetate **12** were done on the basis of their specific rotation values and compared with literature values. In addition, these data were confirmed with the empirical Kazlauskas rule. This rule shows the enantiopreference of the esterification of secondary alcohols by lipase and suggests the attribution of the absolute configuration of products. The absolute configurations of (*R*)-acetates **10**–**11** were suggested by the empirical Kazlauskas rule [5,24].

The enantiomeric excesses of alcohol **1** and acetates (**7**, **9**–**11**) were determined by chiral column chromatography. The *rac*-alcohols **3**–**6** did not show enantioseparation on the chiral column chromatography used. In these cases, these alcohols were derivatized with pyridine/anhydride acetic to produce the corresponding acetates **9**–**12**. The enantiomeric excesses of its acetate derivatives were determined by gas chromatography with an FID detector using chiral column (Table 1).

## 3. Experimental

### 3.1. General

**Scheme 2:**
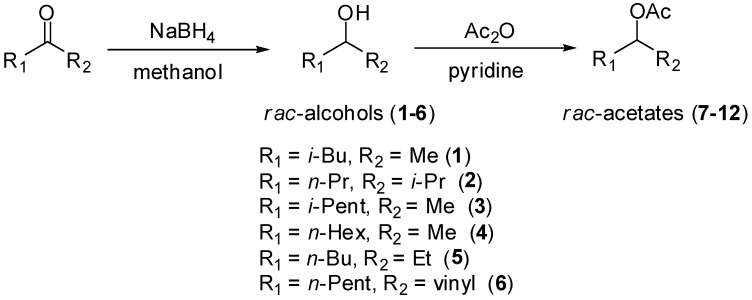
Preparations of (±)-alcohols **1**–**6** and (±)-acetates **7**–**12**.

Vinyl acetate, aliphatic ketones (4-methylpentan-2-one, 2-methylhexan-3-one, 5-methylhexan-2-one, 5-methylhexan-2-one, 5-methylhexan-2-one, oct-1-en-3-one), Ac_2_O, pyridine, NaBH_4_ were purchased from Sigma-Aldrich (St. Louis, MO, USA), and the solvents (AcOEt, hexane) were purchased from Synth (São Paulo, SP, Brazil). Tecnal TE-421 (São Paulo, SP, Brazil) or Superohm G-25 (Piracicaba, SP, Brazil) orbital shakers were employed in the KR reactions. The purification of the products was carried out by column chromatography (CC) over silica gel (230–400 mesh) eluted with mixtures of hexane and AcOEt. The collected fractions were monitored by TLC on aluminum-backed pre-coated silica gel 60 F_254_ (Sorbent-Technologies) layers eluted with hexane and AcOEt, and visualized by spraying with *p*-anisaldehyde/H_2_SO_4_ reagent followed by heating at *ca.* 60°. Syntheses of (±)-aliphatic secondary alcohols **1**–**6** and acetates **7**–**12** are describes in Scheme 2. NMR spectra were recorded on a Bruker AC-200 spectrometer using CDCl_3_ as solvent and TMS as internal calibration. 

#### 3.1.1. Preparation of (±) Aliphatic Secondary Alcohols

Racemic secondary alcohols **1**–**6** were prepared by reducing commercial ketones (4-methylpentan-2-one, 2-methylhexan-3-one, 5-methylhexan-2-one, 5-methylhexan-2-one, 5-methylhexan-2-one, oct-1-en-3-one) using sodium borohydride in methanol. The ketones (5 mmol), NaBH_4_ (5.5 mmol) and methanol (10 mL) were added to a 25 mL flask equipped with a magnetic stirrer. The mixtures were stirred for 4 h at r.t. (Scheme 1). Next, the reactions were quenched by the addition of water (1 mL), the methanol was removed under vacuum and the residue was extracted with ethyl acetate (3 × 20 mL). The combined organic phases were dried over MgSO_4_ and then filtered. The organic solvent was evaporated under reduced pressure and the residue was purified by silica gel column chromatography using hexane and ethyl acetate as eluent to produce racemic alcohols **1**–**6** in excellent yields (**1**, 70%; **2**, 95%; **3**, 92%; **4**, 97%; **5**, 94%; **6**, 93%). The spectroscopic data (^1^H-NMR, MS and IR) of alcohols **1**, **2**, **4** and **6** agreed with the data reported in the literature [22,25,26,27,28].

*5-Methylhexan-2-ol* (**3**): ^1^H-NMR (300 MHz, CDCl_3_), δ (ppm): 3.74 (1H, m), 1.72 (1H, br, OH), 1.05–1.58 (5H, m), 1.16 (3H, d, *J* = 6.0 Hz), 0.87 (6H, d, *J* = 6.0 Hz). ^13^C-NMR (75 MHz, CDCl_3_), δ (ppm): 68.4 (CH), 37.1 (CH_2_), 34.9 (CH_2_), 28.0 (CH), 23.4 (CH_3_), 22.6 (CH_3_), 22.5 (CH_3_).

*Heptan-3-ol* (**5**): ^1^H-NMR (300 MHz, CDCl_3_), δ (ppm): 3.47 (1H, m), 1.61 (1H, br, OH), 1.55–1.15 (8H, m), 0.92–0.83 (6H, br t, CH_3_-CH_2_). ^ 13^C-NMR (75 MHz, CDCl_3_), δ (ppm): 73.2 (CH), 36.6 (CH_2_), 30.1 (CH_2_), 27.8 (CH_2_), 22.7 (CH_2_), 14.0 (CH_3_), 9.8 (CH_3_).

#### 3.1.2. Preparation of (±) Aliphatic Secondary Acetates

Alcohols **1**–**6** (3.0 mmol), pyridine (0.5 mL, 6.2 mmol) and Ac_2_O (0.5 mL, 5.3 mmol) were added to a 25 mL flask equipped with a magnetic stirrer. The mixture was stirred for 24 h at r.t. (Scheme 2). The reactions were quenched by the addition of 10% HCl (2 mL), and the organic phase was extracted with ethyl acetate (3 × 20 mL). The combined organic phases were dried over MgSO_4_ and then filtered. The organic solvent was evaporated under reduced pressure and the residue was purified by silica gel column chromatography using hexane and ethyl acetate as eluent to give racemic acetates **7**–**12** in good to high yields (**7**, 80%; **8**, 87%; **9**, 85%; **10**, 92%; **11**, 89%; **12**, 93%). The spectroscopic data (^1^H-NMR, MS and IR) of acetates **7**, **10** and **12** agreed with the data reported in the literature [22,25,27].

*5-Methylhexan-2-yl acetate* (**9**): ^1^H-NMR (300 MHz, CDCl_3_), δ (ppm): 4.85 (1H, m), 2.00 (3H, s), 1.45–1.55 (3H, m), 1.05–1.20 (1H, overlap), 1.18 (3H, d, *J* = 6.0 Hz), 0.86 (6H, d, *J* = 6.0 Hz). ^13^C-NMR (75 MHz, CDCl_3_), δ (ppm): 170.8, 71.3, 34.4, 33.7, 27.9, 22.5 (2C), 21.3, 20.0. 

*Heptan-3-yl acetate* (**11**): ^1^H-NMR (300 MHz, CDCl_3_), δ (ppm): 4.79 (1H, m), 2.02 (3H, s), 1.61–145 (4H, m), 1.35–1.20 (4H, m), 0.89-0.84 (6H, overlap, CH_3_-CH_2_). ^13^C-NMR (75 MHz, CDCl_3_), δ (ppm): 171.0, 75.5, 33.3, 27.5, 27.0, 22.6, 21.2, 14.0, 9.5.

### 3.2. Biocatalyzed Enzymatic Reactions

Racemic alcohols **1**–**6** (0.5 mmol) were added to a 50 mL Erlenmeyer flask containing 10 mL of hexane (HPLC grade), 0.5 mL of vinyl acetate and 80 mg of immobilized lipase B from *C. antarctica*. The reaction mixture was stirred in an orbital shaker (32 °C, 150 rpm) until the consumption of the reagents (Table 1). The mixture was filtered and the solvent evaporated. The residue was purified by silica gel column chromatography using hexane and AcOEt producing in good yields the enantiopure alcohols **1**, **3**–**6** and acetates **7**, **9**–**12** (Table 1). The progress of the reactions was monitored by collecting samples (0.1 mL) and these were analyzed by GC-FID (1.0 µL) in a chiral capillary column. The products were compared with the previously analyzed racemic mixtures. A similar procedure was repeated to obtain the isolated yield (Table 1). In this case, a 50 mL Erlenmeyer flask was used containing 10 mL of hexane, 1.0 mL of vinyl acetate, 100 mg of lipase CALB and 2.0 mmol of racemic alcohols **1**, **3**–**6**.

### 3.3. GC-FID Analyses

**Figure 1:**
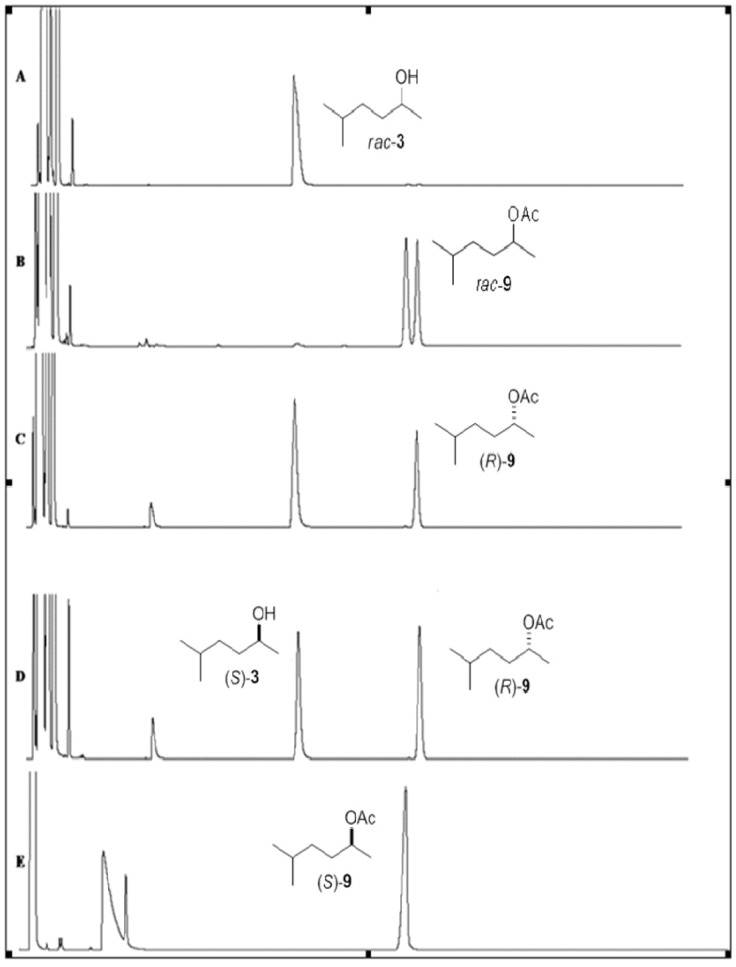
Chiral analyses obtained by GC-FID chromatograms. (**A**) Racemic alcohol (±)-**3**. (**B**) Racemic acetate (±)-**9**. (**C**) Chromatograms of the kinetic resolution of *rac*-**3** by lipase CALB (20 min). (**D**) Chromatograms of the kinetic resolution of *rac*-**3** by lipase CALB (40 min). (**E**) Enantiopure acetate (*S*)-**9** obtained after acetylating of alcohol (*S*)-**3** by Ac_2_O and py.

**Figure 2:**
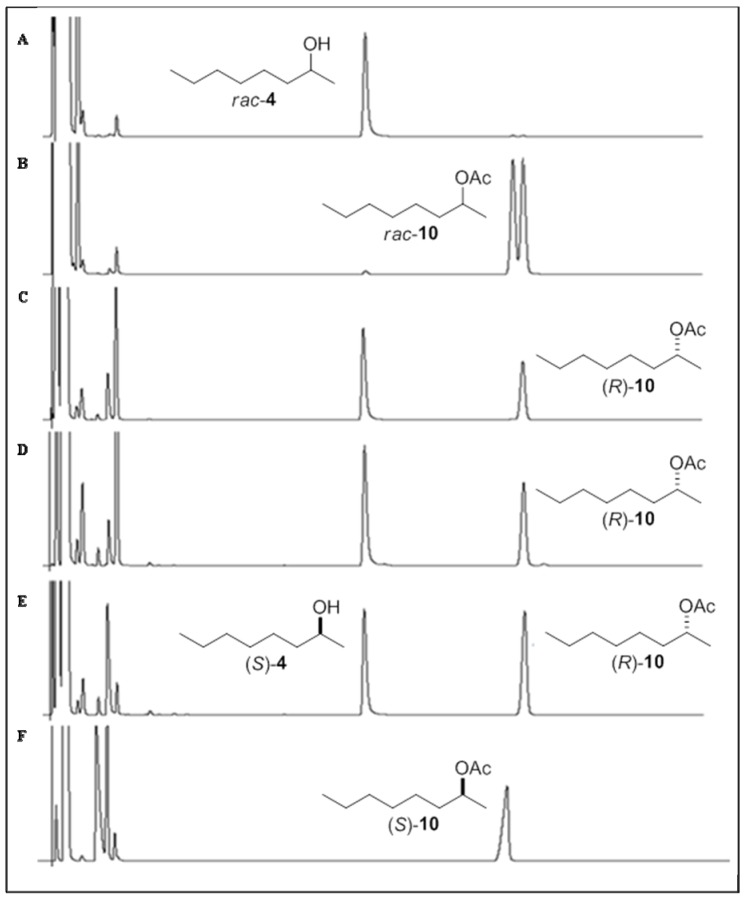
Chiral analyses obtained by GC-FID chromatograms. (**A**) Racemic alcohol (±)-**4**. (**B**) Racemic acetate (±)-**10**. (**C**) Chromatograms of the kinetic resolution of *rac*-**4** by lipase CALB (20 min). (**D**) Chromatograms of the kinetic resolution of *rac*-**4** by lipase CALB (40 min). (**E**) Chromatograms of the kinetic resolution of *rac*-**4** by lipase CALB (60 min). (**F**) Enantiopure acetate (*S*)-**10** obtained after acetylating of alcohol (*S*)-**4** by Ac_2_O and py.

**Figure 3:**
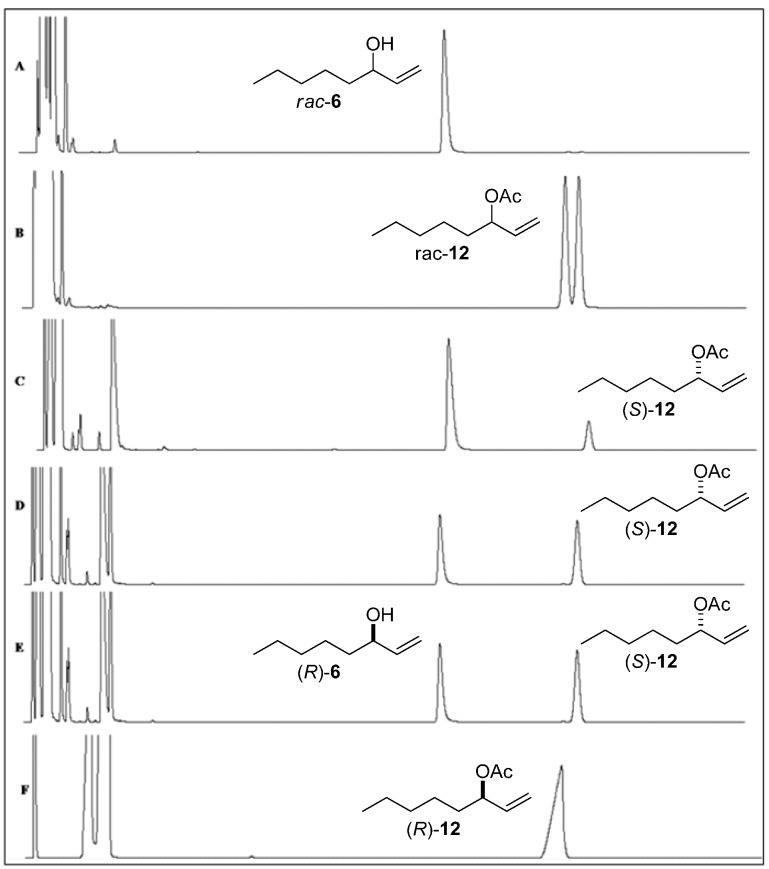
Chiral analyses obtained by GC-FID chromatograms. (**A**) Racemic alcohol (±)-**6**. (**B**) Racemic acetate (±)-**12**. (**C**) Chromatograms of the kinetic resolution of *rac*-**6** by lipase CALB (20 min). (**D**) Chromatograms of the kinetic resolution of *rac*-**6** by lipase CALB (40 min). (**E**) Chromatograms of the kinetic resolution of *rac*-**6** by lipase CALB (60 min). (**F**) Enantiopure acetate (*R*)-**12** obtained after acetylating of alcohol (*R*)-**6** by Ac_2_O and py.

The reaction products were analyzed using a Hewlett Packard (Palo Alto, CA, USA) model HP-5890 gas chromatograph and using a Shimadzu GC 241 gas chromatograph equipped with an AOC 20i auto injector equipped with a Varian CP**-**Chiralsil**-**DEX β-Cyclodextrin column (25 m × 0.25 mm i.d.; 0.39 µm). The programs used for the GC-FID analyses of *rac*-alcohols **1**–**6** and *rac*-acetates **7**–**12** are described in Table 2. The injector and detector were maintained at 200 °C, the split ratio of the injector was 1:20, and the carrier gas was N_2_ at 60 kPa. The *ee* values of alcohols and acetates were determined by GC-FID analyses (Figure 1，Figure 2，Figure 3 and Table 2).

### 3.4. Assignment of the Absolute Configuration

The optical rotation of the products from the biocatalytic reaction was measured in a Perkin-Elmer (Waltham, MA, USA) model 241 polarimeter using a 1 dm cuvette and referenced to the Na–D line. The absolute configurations of compounds (**1**, **4**–**6**, **12**) were determined comparing the specific rotation signs measured for the products with that reported in the literature (Table 3) [14,25,26,27,28,29]. The compounds **3**, **7**, **9**–**11** were suggested by empirical rule of Kazlauskas [5,24].

**Table 2:** Programs used for identification of the alcohols **1**–**6** and acetates **7**–**12** by GC-FID analyses ^a^.

Compounds	T_i_ (°C)	T_f_ (°C)	t_i_ (min)	t_f_ (min)	r (°C /min)	t_r_ (min)
**1**	45	60	2	1	0.2	(*R*)-20.0; (*S*)-21.5
**2**	50	100	2	1	1	(*R*)-9.0; (*S*)-9.0
**3**	65	85	2	1	1	(*R*)-9.0; (*S*)-9.0
**4**	95	110	2	1	1	(*R*)-6.6; (*S*)-6.6
**5**	65	85	2	1	1	(*R*)-10.4; (*S*)-10.4
**6**	85	100	2	1	1	(*R*)-9.3; (*S*)-9.3
**7**	60	100	2	5	5	(*R*)-14.5; (*S*)-22.0
**8**	50	100	2	1	1	(*R*)-12.2; (*S*)-12.5
**9**	65	85	2	1	1	(*R*)-12.2; (*S*)-12.5
**10**	95	100	2	1	1	(*R*)-8.9; (*S*)-9.1
**11**	65	85	2	1	1	(*R*)-13.8; (*S*)-14.1
**12**	85	100	2	1	1	(*R*)-11.6; (*S*)-11.8

^a^ Chiral column: CP**-**Chiralsil**-**DEX β-Cyclodextrin (25 m × 0.25 mm i.d.; 0.39 µm); T_i_: initial temperature; T_f_: final temperature; t_i_: initial time; t_f_: final time; r: rate; t_r_: retention time; min: minutes.

**Table 3:** Experimental data of optical rotations of the aliphatic secondary alcohols and acetates obtained by lipase B from *Candida antarctica*.

Compounds	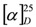 experimental	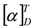 literature
(*S*)-4-methylpentan-2-ol (**1**)	+4.40 (*c* 0.4; CHCl_3_)	*R*-enantiomer: −3.97 (neat) [29]
		*R*-enantiomer: −18.4 (*c* 1.2; EtOH)[27]
(*S*)-5-methylhexan-2-ol (**3**)	+8.68 (*c* 0.5; CHCl_3_)	
(*S*)-octan-2-ol (**4**)	+7.90 (*c* 0.6; CHCl_3_)	*S*-enantiomer: +10.3 (*c* 2.0; EtOH) [25]
		*S*-enantiomer: +8.7 (*c* 1.0; CHCl3) [26]
(*S*)-heptan-3-ol (**5**)	+8.24 (*c* 0.2; CHCl_3_)	*R*-enantiomer: −9.2 (*c* 7–8 g/100mL; CHCl_3_) [30]
		*S*-enantiomer: +9.2 (*c* 7–8 g/100mL; CHCl_3_) [30]
(*R*)-oct-1-en-3-ol (**6**)	−8.06 (*c* 0.8; CHCl_3_)	*S*-enantiomer: +7.0 (*c* 1.0; pentane) [22]
		*S*-enantiomer: +7.16 (*c* 1.38; pentane) [14]
(*R*)-4-methylpentan-2-yl acetate (**7**)	−7.10 (*c* 0.8; CHCl_3_)	*R*-enantiomer: −21.8 (*c* 2.0; CHCl_3_) [27]
(*R*)-octan-2-yl acetate (**10**)	−2.96 (*c* 0.5; CHCl_3_)	
(*R*)-heptan-3-yl acetate (**11**)	+1.79 (*c* 0.7; CHCl_3_)	
(*S*)-oct-1-en-3-yl acetate (**12**)	−12.07 (*c* 0.9; CHCl_3_)	*S*-enantiomer: −11.5 (*c *2.8; CHCl_3_) [22]

### 3.5. Enantiomeric Ratio (E)

The enantiomeric ratio (*E*) for the kinetic resolutions of racemic alcohols was calculated for catalysed reactions by equations formulated by Sih and coworkers [31]. The calculation of the selectivity of the kinetic resolution expressed as enantiomeric ratio (*E*) for irreversible reactions was obtained by a computer program described by Faber [32].

## 4. Conclusions

In summarized, we have reported the chemoenzymatic transesterification of aliphatic *rac*-secondary alcohols under mild conditions. The lipase CALB-catalysed enantioselective esterification reaction afforded the alcohols (**1**, **3**–**6**) and acetates (**7**, **9**–**12**), in accordance with the Kazlauskas rule, yielding high enantiomeric excesses (>99% *ee*).Various substituent groups (isobutyl, isopentyl, *n*-butyl, *n*-pentyl, *n*-hexyl, ethyl, methyl, vinyl) attached to the stereogenic center were efficiently accepted by lipase CALB.
